# Carbon wrapped hierarchical Li_3_V_2_(PO_4_)_3_ microspheres for high performance lithium ion batteries

**DOI:** 10.1038/srep33682

**Published:** 2016-09-21

**Authors:** Shuquan Liang, Qinguang Tan, Wei Xiong, Yan Tang, Xiaoping Tan, Linjun Huang, Anqiang Pan, Guozhong Cao

**Affiliations:** 1 School of Materials Science & Engineering, Central South University, Hunan, 410083, China; 2 Department of Materials, Imperial College London, London, UK; 3 Department of Materials Science & Engineering, University of Washington, Seattle, 98195, WA, USA

## Abstract

Nanomaterials are extensively studied in electrochemical energy storage and conversion systems because of their structural advantages. However, their volumetric energy density still needs improvement due to the high surface area, especially the carbon based nanocomposites. Constructing hierarchical micro-scaled materials from closely stacked subunits is proposed as an effective way to solve the problem. In this work, Li_3_V_2_(PO_4_)_3_@carbon hierarchical microspheres are prepared by a solvothermal reaction and subsequent annealing. Hierarchical Li_3_V_2_(PO_4_)_3_ structures with different subunits are obtained with the aid of polyvinyl pyrrolidone (PVP). Moreover, excessive PVP interconnect and form PVP-based hydrogels, which later convert into conductive carbon layer on the surface of Li_3_V_2_(PO_4_)_3_ microspheres during the annealing process. As a cathode material for lithium ion batteries, the 3D carbon wrapped Li_3_V_2_(PO_4_)_3_ hierarchical microspheres exhibit high rate capability and excellent cycling stability. The electrode has the capacity retention of 80% after 5000 cycles even at 50C.

Carbon based nanocomposites have been broadly studied in electrochemical energy storage and conversion applications due to their synergistic effects between active materials and carbon^1^,^2^,^3^,^4^,^5^. To date, different approaches have been reported to synthesize carbon based nanocomposites, such as templating against carbon nanotubes and graphene^6^,^7^,^8^,^9^, surface coating^10^,^11^,^12^, and *in situ* conversion from organic precursors^13^,^14^. In general, the obtained carbon based nanocomposites are of high quality and show enhanced electrochemical properties, including high capacity, good rate capability and cycling stability^15^,^16^,^17^,^18^. However, the loading of the active materials in the carbon based composites is quite low^19^,^20^,^21^. For instance, the mass loading of sulfur in the carbon and sulfur composite is usually less than 70%, though extremely high specific capacities are often reported^22^. How to improve the mass loading of active material in the carbon-based composites is the key to high volumetric energy density^23^,^24^. Recently, three-dimensional (3D) hierarchical microstructures assembled from nanoscaled subunits have been greatly investigated for lithium ion batteries because of their good electrochemical properties and high energy packing density^25^,^26^,^27^,^28^. The nanoscaled subunits provide shorter lithium diffusion distance and larger contact area between electrode and electrolyte, and the self-assembled hierarchical microstructures improve the energy packing density^29^,^30^. Therefore, constructing carbon and active material hierarchical structures is believed a promising way to achieve good electrochemical performance and desired energy density^31^,^32^,^33^.

Lithium transition metal phosphates are widely used as cathode materials in lithium ion batteries for high power electrical vehicles because of their good safety and cycling stability^34^,^35^. However, the required high temperature (>500 °C) synthesis process brings the difficulties of controlling the particle size, which is closely related with good electrochemical properties^36^,^37^. Therefore, novel synthesis routes are investigated to solve this problem. From the perspective of Li_3_V_2_(PO_4_)_3_, Pan *et al*.^38^ reported the fabrication of Li_3_V_2_(PO_4_)_3_/carbon composite by absorbing the liquid precursors into mesoporous carbon framework with subsequent annealing, which successfully reduced the particle growth and improved the rate capability of the Li_3_V_2_(PO_4_)_3_ electrode. Wei *et al*.^39^ employed a hydrothermal-pyrolytic process to fabricate Li_3_V_2_(PO_4_)_3_/C hierarchical nanowires, which exhibit superior cycling stability with a capacity retention of 80% after 3000 cycles at 5C rate. Zhou *et al*.^40^ reported the synthesis of Li_3_V_2_(PO_4_)_3_ nanoparticles within the three-dimensional hierarchical carbon foam, exhibiting outstanding rate capability. However, the volumetric energy density of most reported lithium transition metal phosphate electrodes needs to be improved^41^,^42^,^43^.

In this work, carbon wrapped Li_3_V_2_(PO_4_)_3_ hierarchical microspheres from close stacked nanosheets are prepared by the facile solvothermal process with the aid of PVP. PVP plays a vital role in the formation of hierarchical microspheres and serves as the starting reagent for PVP-based hydrogels, which is carbonized to form the wrapping carbon layer on Li_3_V_2_(PO_4_)_3_ microspheres. As cathode materials for lithium ion batteries, the hierarchical Li_3_V_2_(PO_4_)_3_@carbon microspheres exhibit excellent rate capability and cycling stability.

## Materials and Methods

### Materials Synthesis

In a typical synthesis, V_2_O_5_ (144 mg) and oxalic acid in a molar ratio of 1:3 were dissolved into 12 mL of deionized water at 70 °C to form the VOC_2_O_4_ solution, which was later poured into a 100 mL Teflon container. Then, stoichiometric amount of NH_4_H_2_PO_4_, Li_2_CO_3_ and 2.5 g of polyvinyl pyrrolidone (PVP, molecular weight: 58,000) were added into the solution under magnetic stirring for 30 minutes. After that, 60 mL of isobutanol was added and stirred for another 1 h. The container was sealed in an autoclave and kept at 180 °C for 24 h. After cooling down to room temperature naturally, brown colored precipitate was collected and dried before annealing at 800 °C for 8 h in H_2_ /Ar (5:95, v/v) atmosphere to obtain carbon wrapped hierarchical Li_3_V_2_(PO_4_)_3_ (CW-LVP) microspheres. In order to study the formation process of the hierarchical structures, different amount of water (8, 10, and 12 ml) and PVP (0, 1.5 and 2 g) were used during the solvothermal fabrication process. Moreover, the time-dependent experiments (2, 6, 24 and 48 h) were carried out to study the structure evolution.

### Materials Characterization

Crystallographic phases of all the products were investigated by powder X-ray diffraction (XRD, Rigaku D/max2500) with Cu Kα (λ = 1.5406 Å) radiation. The morphologies of the samples were examined by field-emission scanning electron microscopy (SEM, FEI Nova NanoSEM 230) and transmission electron microscopy (TEM; JEOL-JEM-2100F transmission electron microscope). A combined Differential Scanning Calorimetry (DSC)/Thermogravimetric Analysis (TGA) instrument (Netzsch STA449C, Germany) was used to study the reactions during the annealing process and measure the carbon content in CW-LVP. Raman spectra were obtained using a Renishaw INVIA micro-Raman spectroscopy system. Nitrogen adsorption-desorption measurements were conducted at 77K (NOVA 4200e, Quantachrome Instruments).

### Electrochemical Measurements

The working cathode slurry was prepared by dispersing the CW-LVP, acetylene black and poly-(vinylidene fluoride) (PVDF) binder in the N-methylpyrrolidone solution with a weight ratio of 70:20:10. The slurry was painted on the aluminum foil and dried in a vacuum oven at 110 °C for 12 h. The half-cell assembly was carried out in a glove box filled with ultrahigh pure Argon using lithium foil as the anode, and 1.0 M LiPF_6_ in ethyl carbonate/dimethyl carbonate (1:1 v/v ratio) as the electrolyte. Cyclic voltammetry (CV) measurements were performed on an electrochemical workstation (CHI604E, China). The galvanostatic charge/discharge performances of the electrodes were measured at room temperature using a Land Battery Tester (Land CT 2001A, China).

## Results and Discussion

**Figure 1:**
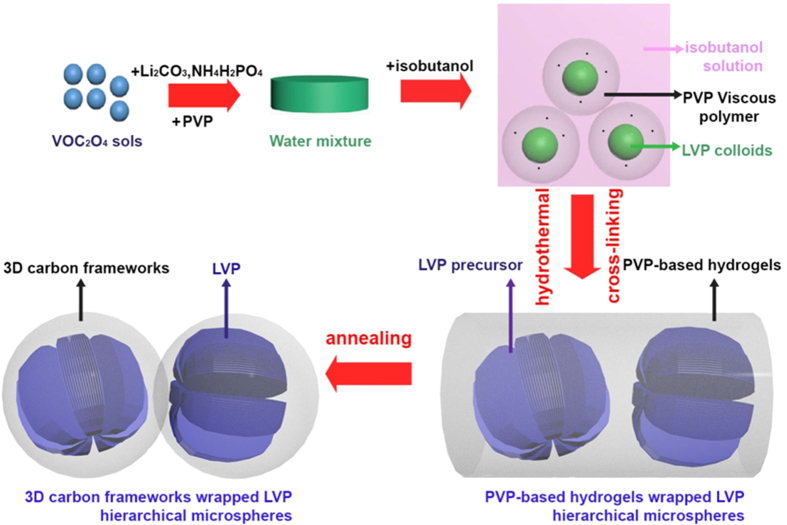
Schematic illustration of the fabrication steps and formation mechanism of the 3D carbon frameworks wrapped Li_3_V_2_(PO_4_)_3_ nanosheets-assembled microspheres.

Figure 1 schematically illustrates the fabrication process of carbon wrapped Li_3_V_2_(PO_4_)_3_ (CW-LVP) microspheres. Firstly, the Li_3_V_2_(PO_4_)_3_ precursor colloids were synthesized using VOC_2_O_4_, Li_2_CO_3_, NH_4_H_2_PO_4_ and PVP. Then isobutanol was added into the colloids to form a two phase solution, where the PVP viscous polymers wrapped on the surface of the colloids. After solvothermal treatment, the hierarchical Li_3_V_2_(PO_4_)_3_ precursor microspheres were fabricated. Meanwhile, the PVP viscous polymers that attached tightly to the surface of the Li_3_V_2_(PO_4_)_3_ precursor particles convert into PVP-based hydrogels. The picture of the solvothermal product is shown in Supplementary Figure S1. Large amount of PVP-based hydrogels are clearly presented in brown color. These PVP-based hydrogels wrapped Li_3_V_2_(PO_4_)_3_ precursor microspheres were annealed to form carbon wrapped Li_3_V_2_(PO_4_)_3_ microspheres. Supplementary Figure S2 compares the TG and DSC results of the Li_3_V_2_(PO_4_)_3_ surrounded by PVP-based hydrogels and pure Li_3_V_2_(PO_4_)_3_ precursor. The weight loss of the composite precursor is 40% percent more than pure Li_3_V_2_(PO_4_)_3_ precursor. The extra weight loss is attributed to the decomposition of dried PVP-based hydrogels at about 436 °C.

**Figure 2:**
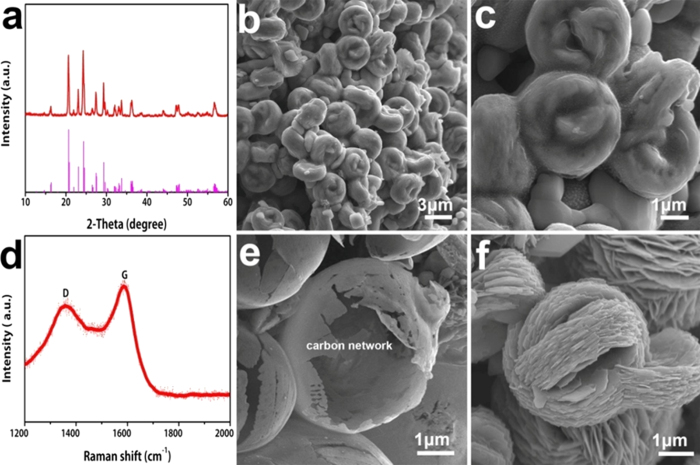
(**a**) XRD patterns of the CW-LVP hierarchical microspheres prepared after annealing. (**b**) and (**c**) SEM images of the CW-LVP hierarchical microspheres. (**d**) Raman spectra of the CW-LVP hierarchical microspheres. (**e**) SEM image of the exterior 3D carbon network after totally removing the LVP microspheres by HCl. (**f**) The SEM image of the interior LVP hierarchical microspheres prepared by removing the PVP-based hydrogels.

Figure 2 shows the structural characterization results of the carbon wrapped hierarchical Li_3_V_2_(PO_4_)_3_ spheres. According to the XRD pattern (Fig. 2a), the obtained material can be assigned to monoclinic Li_3_V_2_(PO_4_)_3_ with a space group *P2*_*1*_*/n* (JCPDS No. 01-072-7074)^42^,^43^,^44^. No other phase is detected, suggesting the high purity of Li_3_V_2_(PO_4_)_3_ samples. The SEM images (Fig. 2b,c) reveal the spherical morphology of the fabricated Li_3_V_2_(PO_4_)_3_, which has an average diameter of 3–4 μm. Moreover, the surface of Li_3_V_2_(PO_4_)_3_ microspheres is coated by a smooth layer (Fig. 2c). Figure 2d shows two broad bands at 1350 and 1590 cm^−1^ on the Raman spectrum of the Li_3_V_2_(PO_4_)_3_ microsphere, which corresponds to the typical D and G bands of carbon. The result indicates the existence of carbon in the composite. Two broad bands at 997 and 1135 cm^−1^ corresponding to the Li_3_V_2_(PO_4_)_3_ phase are also shown in Figure S3. After removing Li_3_V_2_(PO_4_)_3_ in the microspheres by HCl acid leaching, a hollow-structured microsphere is observed (Fig. 2e), which indicates the surface coating layer is composed of carbon. The TEM image (see supplementary information, Figure S4) confirms the existence of carbon layer on the surface of Li_3_V_2_(PO_4_)_3_ microspheres, which is about 30 nm in thickness. The carbon is derived from the PVP-based hydrogels in the calcination process in Argon. According to the thermogravimetric (TG) analysis (Figure S5, supplementary information), the carbon content in the CW-LVP microspheres is 24.6 wt%. The solvothermal precursor product was washed with ethanol eight times to remove the PVP-based hydrogels in order to study the interior structures of Li_3_V_2_(PO_4_)_3_ precursors. The interior Li_3_V_2_(PO_4_)_3_ precursor microspheres are assembled from close stacked nanosheets in parallel (Fig. 2f), which is believed to be helpful to improving the volumetric energy densities of the electrode.

**Figure 3:**
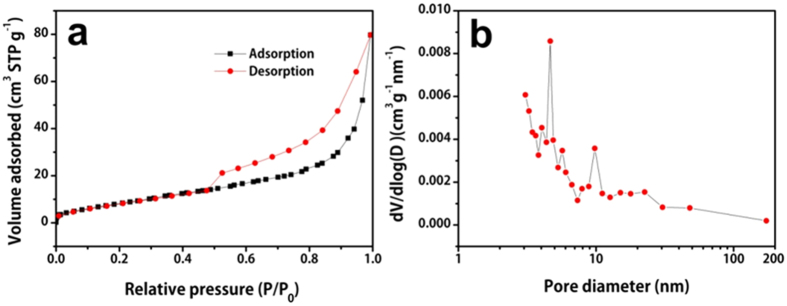
(**a**) Nitrogen adsorption-desorption isotherm and (**b**) the corresponding pore size distributions of the CW-LVP hierarchical microspheres.

Nitrogen adsorption-desorption measurement is carried out to further study the carbon wrapped Li_3_V_2_(PO_4_)_3_ microspheres and the results are shown in Fig. 3. The isothermal curve exhibits a typical IV-type hysteresis, indicating the mesoporous feature of the CW-LVP microspheres. According to Brunauer-Emmett-Teller (BET) method, the surface area of the CW-LVP microspheres is 35.1 m^2^g^−1^. Figure 3b shows the pore size distribution of the CW-LVP by Barret-Joyner-Halenda (BJH) method. The majority of pores are less than 10 nm. It is believed that the pores are largely produced from the surface carbon shell. The porous structure provides easier paths for electrolyte penetration and the reasonable BET surface area gives the sufficient surface contact area between electrode materials and electrolyte.

In order to study the formation of hierarchical microspheres, the solvothermal process is carefully studied, including the addition amount of PVP, isopropanol, and the solvothermal treatment time. The PVP based hydrogels are removed after solvothermal treatment to study the structural evolution of the Li_3_V_2_(PO_4_)_3_ precursor microspheres. The amount of PVP added has a great effect on the morphologies of the solvothermal prepared Li_3_V_2_(PO_4_)_3_ microstructures and the results are shown in Figure S6. No microspheres are formed without PVP in the solvothermal solution. The obtained aggregates are of microscale and have irregular shapes. However, nanosheet assembled hierarchical microspheres are obtained when 1 g PVP is added. By increasing the PVP addition amount to 1.5 g, the nanosheets are closer stacked and form the hierarchical microspheres. Moreover, the usage of isobutanol is vital to build the nanosheet-assembled hierarchical structures. Only microspheres with smooth surface are obtained when no isobutanol is used (see Supplementary Figure S7).

Time-dependent experiment (2, 6, 24 and 48 h) is also carried out to study the structural evolution of the hierarchical precursor microspheres. As shown in Figure S8 (supplementary information), the microspheres with small nanosheet subunits are readily formed after 2 h solvothermal treatment. After 6 h solvothermal treatment, the nanosheets are more clearly detected. By extending the solvothermal time to 24 and 48 h, the nanosheets become larger and more separated. The thickness of the nanosheet is about 20 nm.

**Figure 4:**
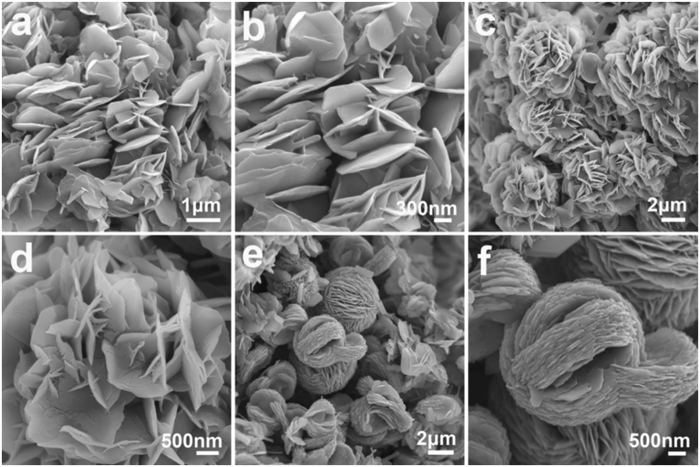
SEM images of the LVP products prepared by adding different amount of water and removing the PVP-based hydrogels. (**a**,**b**) 8 ml, (**c**,**d**) 10 ml, and (**e**,**f**) 12 ml (**e**,**f)**, corresponding to the interior microspheres in CW-LVP).

The volume ratio between isobutanol and water also affects the morphologies of the solvothermal products significantly. Figure 4 shows the SEM images of the Li_3_V_2_(PO_4_)_3_ microspheres prepared from the different volume ratio of isobutanol and water. When 8 mL water is combined with 60 mL isobutanol, the solvothermal products are mainly composed of nanoplates. The lengths of Li_3_V_2_(PO_4_)_3_ nanoplates can be 1–2 μm in width and about 50 nm in thickness (Fig. 4a,b). When the amount of water is increased to 10 mL, the precursor microflowers assembled from petal-like nanosheets are formed (Fig. 4c,d). When increasing the amount of water to 12 mL, hierarchical Li_3_V_2_(PO_4_)_3_ microspheres with close packed parallel nanosheets can be obtained (Fig. 4e,f).

**Figure 5:**
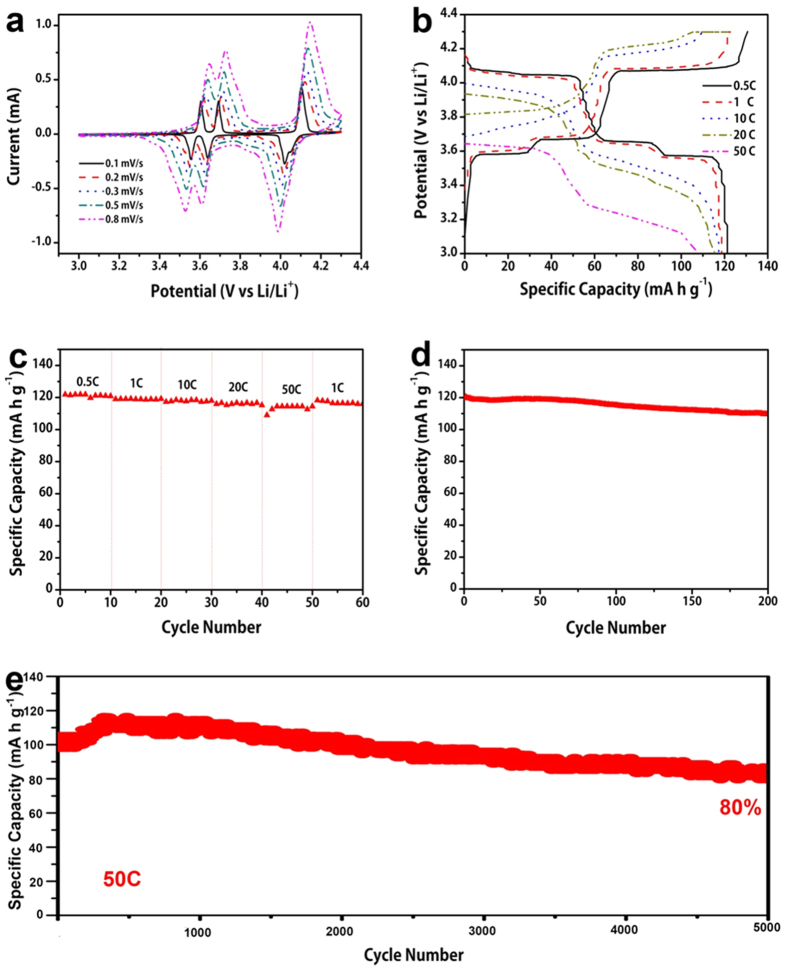
(**a**) Typical cyclic voltammograms (CV) curves of the CW- LVP spheres cathode at different scan rates; (**b**) Discharge-charge profiles of the CW- LVP spheres cathode at different current rates; (**c**) Rate performance of the CW- LVP spheres cathode; (**d**) Cycling performance of the CW- LVP spheres cathode at 1 C; (**e**) Long cycling performance of the CW- LVP spheres in the voltage of 3.0–4.3 V at high current density of 50 C.

The carbon wrapped Li_3_V_2_(PO_4_)_3_ hierarchical microspheres were assembled into coin cells to measure their electrochemical performances. The mass loading density of the CW-LVP electrode is about 1 mg cm^−2^. Figure 5a shows the cyclic voltammograms (CVs) curves of the CW-LVP electrodes in the voltage range of 3.0–4.3 V vs. Li/Li^+^ with different scanning rates. Three intensive pairs of redox peaks are detected on the CV curves at various scan rates, suggesting the good reversibility and stability of the CW-LVP electrode. The detection of multiple peaks at 3.63, 3.72 and 4.14 V during the anodic scan of 0.1 mV s^−1^ show that the multi-step lithium extraction process and the phases change from Li_3_V_2_(PO_4_)_3_ to Li_2.5_V_2_(PO_4_)_3_, Li_2_V_2_(PO_4_)_3_ and to LiV_2_(PO_4_)_3_, respectively^37^,^38^,^39^,^40^. And the three cathodic peaks at 3.99, 3.62, and 3.54 V are attributed to lithium insertion process, and the phases change in reverse, respectively. The small peak shift at high scan rates suggests the low polarization of the electrode materials.

Figure 5b shows the charge-discharge profiles of the CW-LVP electrodes at various rates (Here 1 C corresponds to 133 mA g^−1^) in the voltage range of 3.0–4.3 V. The discharge plateaus at 3.99, 3.62 and 3.54 V and charge plateaus at 4.14, 3.72 and 3.63 V at 0.5 C are clearly observed, demonstrating the multi-step Li^+^ ions intercalation/de-intercalation process. Result match well with the CV curves. The discharge/charge profiles almost overlapped before 1 C rate. The main plateaus can be clearly presented even at 10, 20 and 50 C. The initial charge and discharge capacities at 0.5 C are 130.7 mA h g^−1^ and 121.3 mA h g^−1^, respectively. And the corresponding initial coulombic efficiency at 0.5 C is about 92.8%. The capacity loss may be attributed to the formation of SEI layer on the surface of the electrode materials. Figure 5c shows the rate performance of the electrode materials. The CW-LVP microspheres have specific capacities of 122, 120 and 115 mA h g^−1^ at 0.5, 1, and 20 C, respectively. After charging at the rate of 20 C, a specific discharge capacity of 110 mA h g^−1^ can be achieved even at discharging rate of 50 C, which is 90% of the capacity at 0.5 C. When the current is reset to 1 C, a capacity of 120 mA h g^−1^ can be restored. Results demonstrate the excellent rate capability of the electrode materials. Figure 5d shows the cycling performance of the CW-LVP electrode at 1 C. The initial specific discharge capacity of CW-LVP is 120.5 mA h g^−1^. After 200 cycles, it retains a capacity of 110.1 mA h g^−1^, giving a capacity retention of 91.4%. The long-term cycling stability of the electrode at 50 C is also evaluated and the result is shown in Fig. 5e. The CW-LVP cathode exhibits a high initial discharge capacity of 105.3 mA h g^−1^ and gradually increases to 112.8 mA h g^−1^ after 500 cycles. The continuous capacity increase in the initial cycles can be attributed to the electrode wettability by the electrolyte, particularly at high rates^45^. After 5000 cycles, the CW-LVP electrode still retains a stable capacity of 85 mA h g^−1^, giving a capacity retention of 80.7%. The result demonstrates the superior cycling stability of the electrode. The electrochemical impedance spectrum (Supplementary Figure S9) simulation result shows that the charge transfer resistance of the CW-LVP electrode is only 85.5 Ohm, which is much lower than 500 Ohm of the LVP particle electrode without using PVP in the fabrication process. The superior rate performance and cycling stability is much better than the previously reported electrodes (see Supplementary Table S1)^14^,^37^,^38^,^39^,^40^,^44^,^46^,^47^,^48^. Although the carbon content in CW-LVP is a little high for practical application, the bicontinuous carbon in the CW-LVP is considered to be a better conducting material than the common discontinuous acetylene black for cathode. As shown in Supplementary Figure S10, the carbon wrapped LVP microspheres exhibit higher capacity and much better rate capability than the LVP microspheres without carbon layer on their surface. The result demonstrates the significant contribution of the carbon wrapping layer on the electrochemical performance improvement. Some similar structures with lower carbon content can be achieved by changing the amount of PVP or water in the solvothermal process. When the amounts of PVP are decreased to 1.5 and 1.0 g, the carbon content of carbon wrapped LVP microspheres is decreased to 12.6% and 2.8%, respectively (Supplementary Figure S11a). When the amount of distilled water in solvent is decreased to 10 mL, the carbon content of carbon wrapped LVP microflowers is decreased to 9.45% (Supplementary Figure S11b). The excellent electrochemical performance of the CW-LVP electrodes can be attributed to the three dimensional carbon wrapped, nanosheet-assembled hierarchical microspheres: (1) the porous carbon shell can improve the electron transportation and provide the electrolyte penetration path; (2) the nanosheet subunits can reduce the Li^+^ ion diffusion and electron transportation distance; (3) the carbon shell can better keep the structural integrity upon repeated charge/discharge process; (4) the hierarchical microspheres can reduce the self-aggregation upon cycling, thus possessing better cycling stability.

## Conclusions

In summary, three-dimensional carbon wrapped Li_3_V_2_(PO_4_)_3_ hierarchical microspheres are successfully synthesized by the solvothermal method and subsequent annealing process. The formation of the hierarchical precursor microspheres during the solvothermal process is investigated. As a cathode material for lithium ion batteries, carbon wrapped Li_3_V_2_(PO_4_)_3_ microspheres show excellent long-term stability and rate capability. These superior electrochemical performances are attributed to the favorable carbon wrapped hierarchical structures, which ensure the fast lithium ion diffusion, high conductivity and great structural stability.

## Additional Information

**How to cite this article**: Liang, S. *et al*. Carbon wrapped hierarchical Li_3_V_2_(PO_4_)_3_ microspheres for high performance lithium ion batteries. *Sci. Rep.* **6**, 33682; doi: 10.1038/srep33682 (2016).

## Supplementary Material

Supplementary Information
