# Supplementary material for: Carbon wrapped hierarchical Li3V2(PO4)3 microspheres for high performance lithium ion batteries
Source: Sci Rep. 2016 Sep 21;6:33682. doi: 10.1038/srep33682 (PMC5030488; doi:10.1038/srep33682)
Supplement: Supplementary Information [file srep33682-s1.doc]

**Carbon wrapped hierarchical Li_3_V_2_(PO_4_)_3_ microspheres for high performance lithium ion batteries**

*Shuquan Liang,^a^ Qinguang Tan,^a^ Wei Xiong,^c^ Yan Tang,^a^ Xiaoping Tan,^a^ Linjun Huang,^a^ Anqiang Pan,^a,*^and Guozhong Cao ^b,*^*

*^a^School of Materials Science & Engineering, Central South University, Hunan, 410083, China*

*^b^Department of Materials Science & Engineering, University of Washington, Seattle, 98195, WA, USA*

*^c^Department of Materials, Imperial College London, London, UK*

** Corresponding author: pananqiang@csu.edu.cn (A.Q. Pan),* [*gzcao@u.washington.edu*](mailto:gzcao@u.washington.edu) *(G.Z. Cao)*


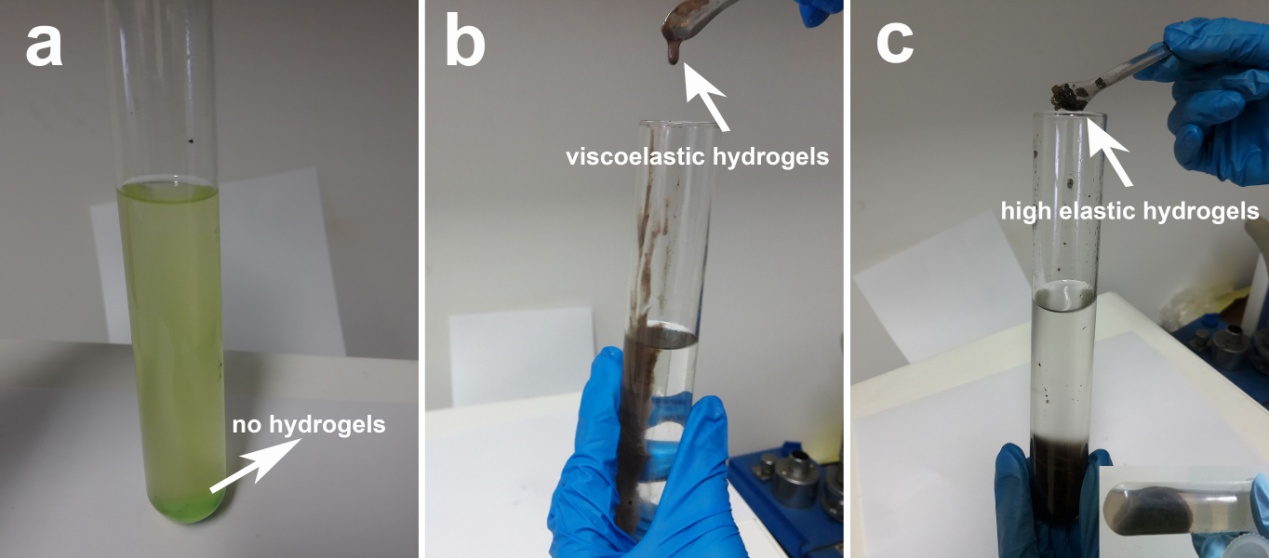


**Figure S1.** Related photographs of the LVP microstructures precursors after the solvothermal process: (**a**) LVP precursors synthesized with 6 mL water, (**b**) CW-LVP microflowers precursor synthesized with 10 mL water and (**c**) CW-LVP microspheres precursor synthesized with 12 mL water.


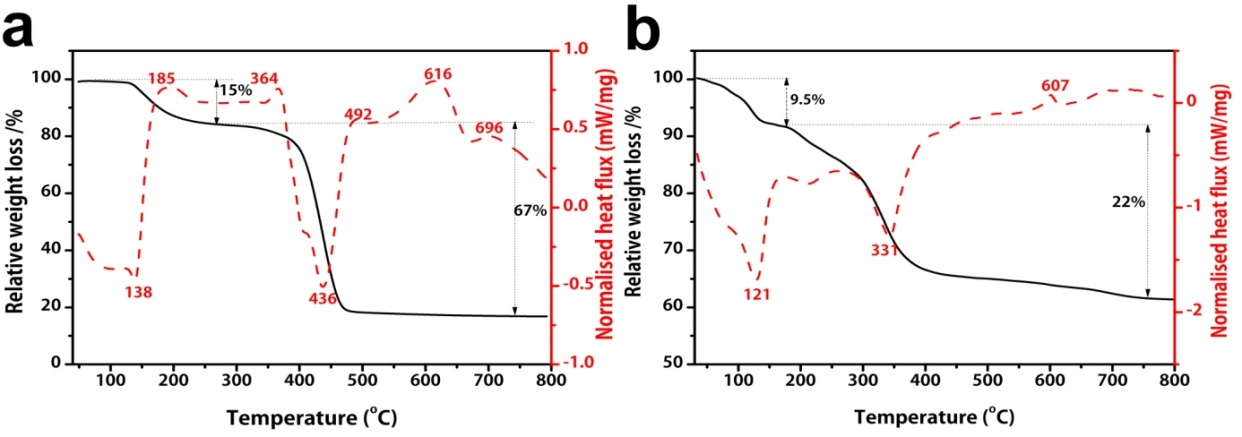


**Figure S2.** TG and DSC curves of the solvothermal synthesized LVP precursors in pure argon atmosphere: (**a**) CW-LVP microspheres precursor and (**b**) no hydrogels wrapping LVP precursor.


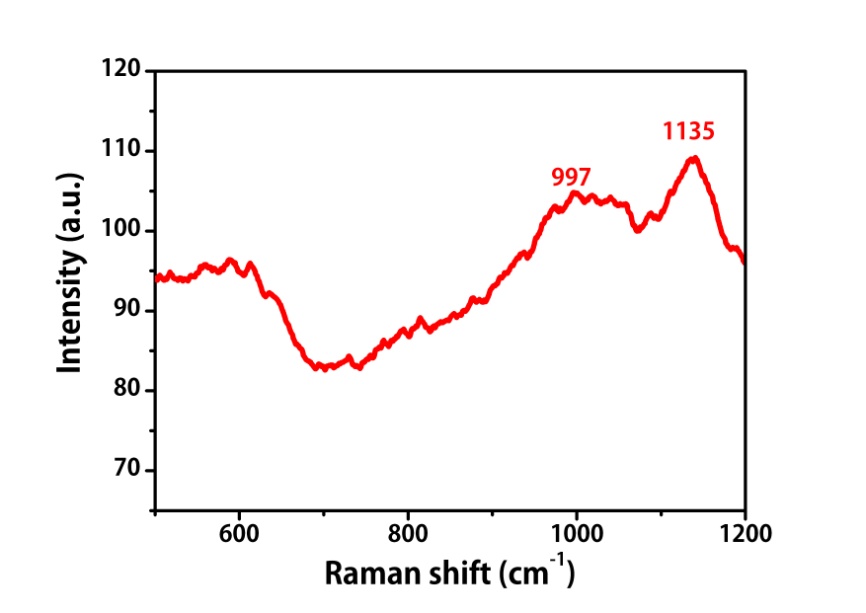


**Figure S3.** Raman spectra of the CW-LVP hierarchical microspheres


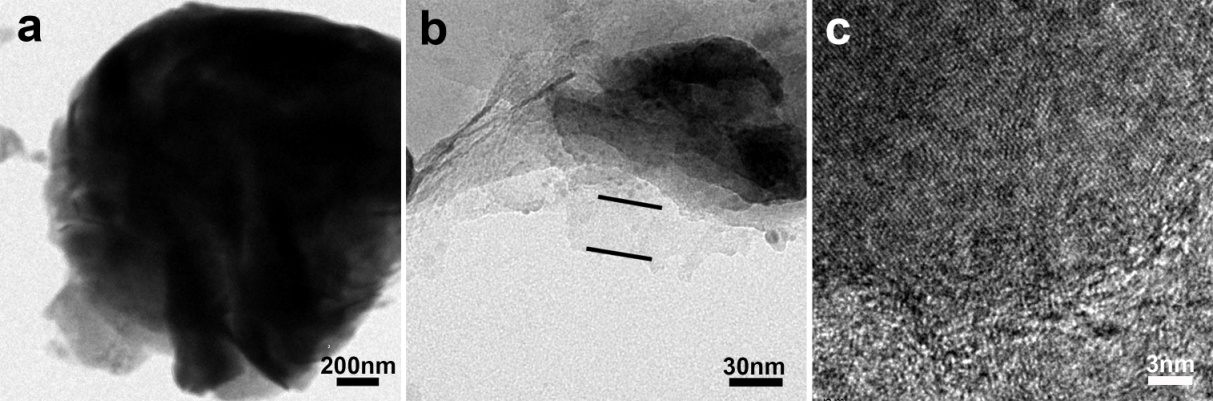


**Figure S4.** TEM images of the as-synthesized CW-LVP microspheres: (**a**) low magnification, (**b**) carbon layer and (**c**) interface between the subunits and the carbon frameworks.

**
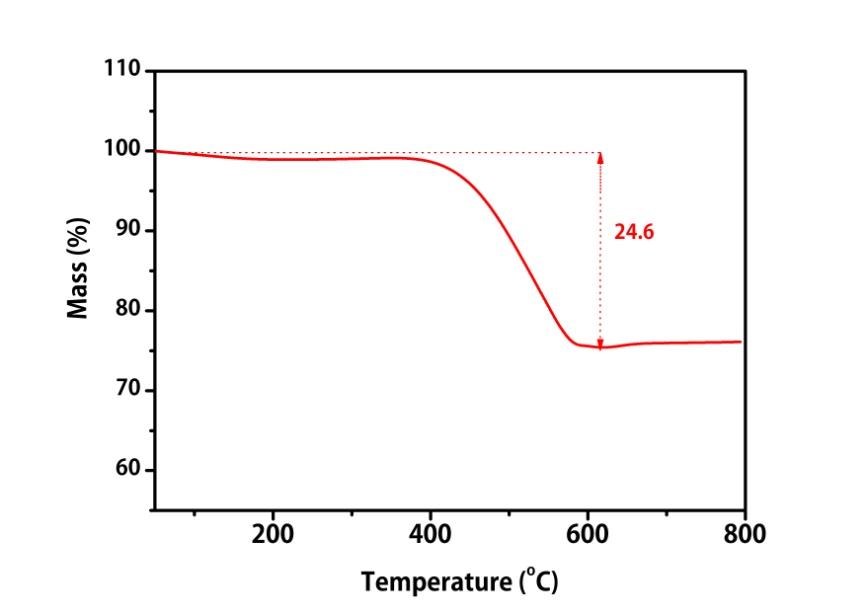
**

**Figure S5.** TG result of the CW-LVP microspheres between room temperature and 800 ^o^C in air using a ramping rate of 10 ^o^C min^-1^.


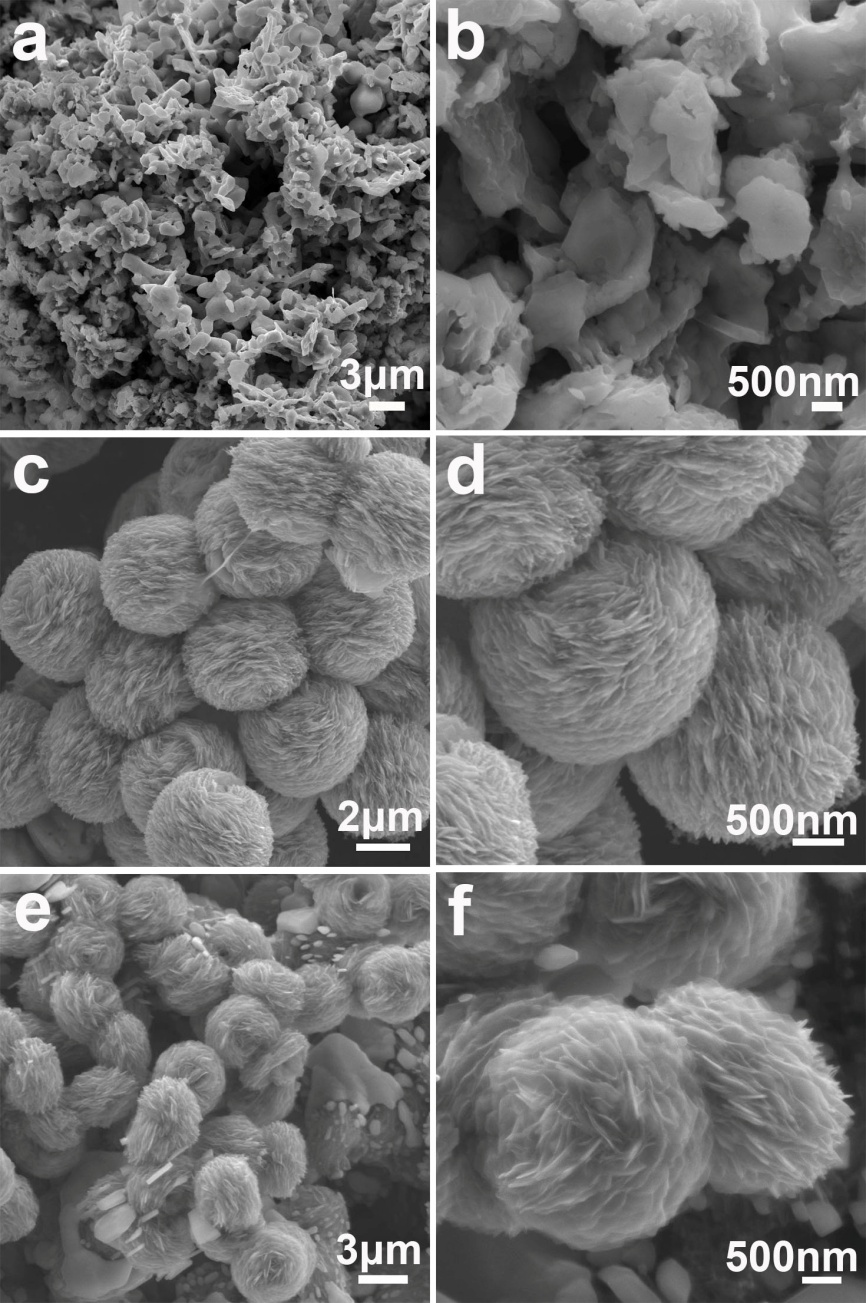


**Figure S6.** SEM images of the LVP microspheres prepared by using different amounts of PVP: (**a**, **b**) 0g, (**c**, **d**) 1.0 g, and (**e**, **f**) 1.5g.


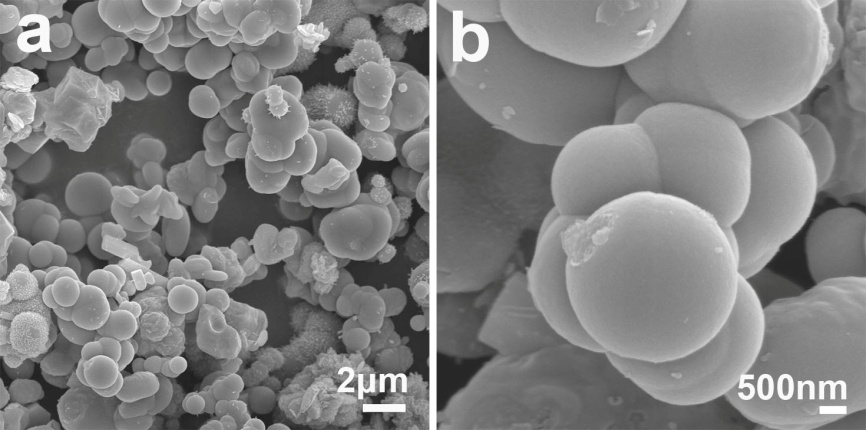


**Figure S7.** FESEM images (a, b) of the LVP precursor synthesized with pure water as the solvent instead of isobutanol-water solution.


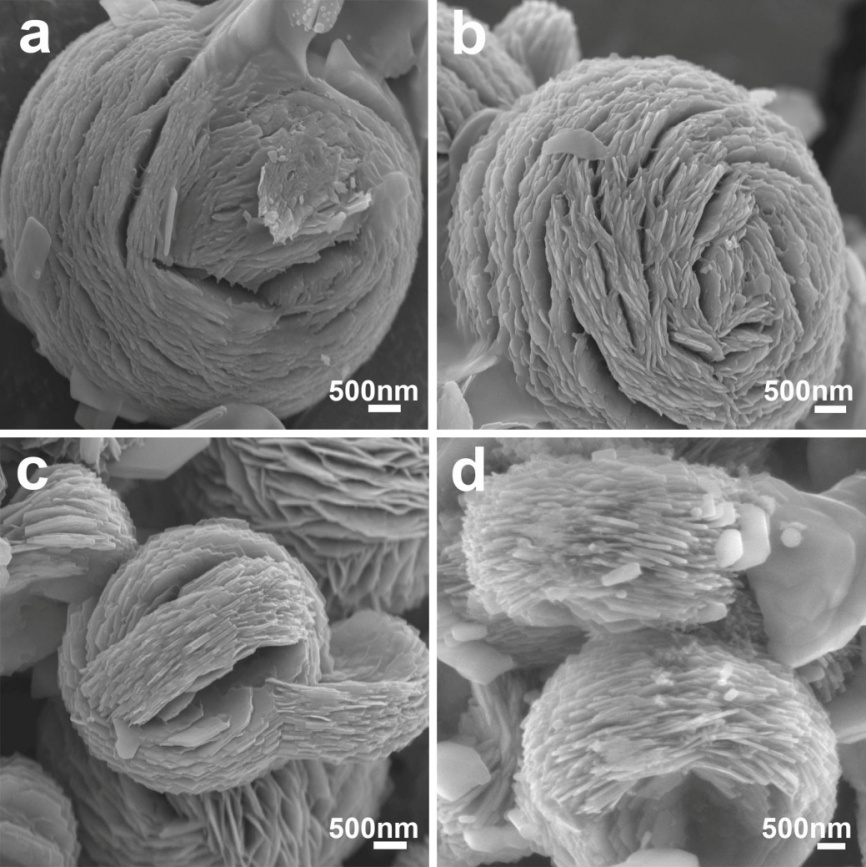


**Figure S8.** FESEM images of the LVP microspheres prepared after different solvothermal treatment durations: (a) 2h, (b) 6h, (c) 24h, and (d) 48h.


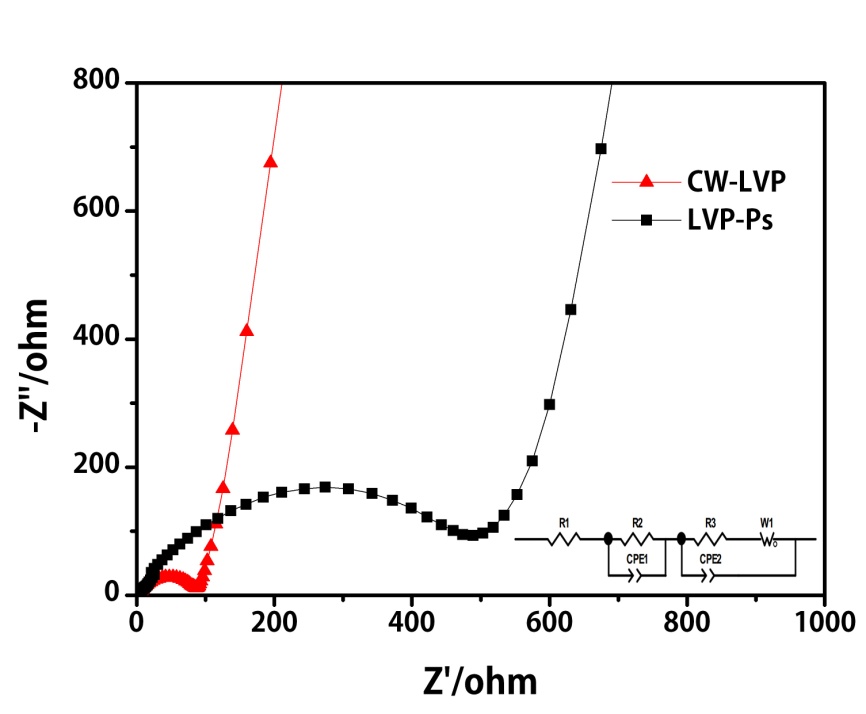


**Figure S9.** Nyquist plots of the CW-LVP hierarchical microspheres and the LVP-Ps electrodes.


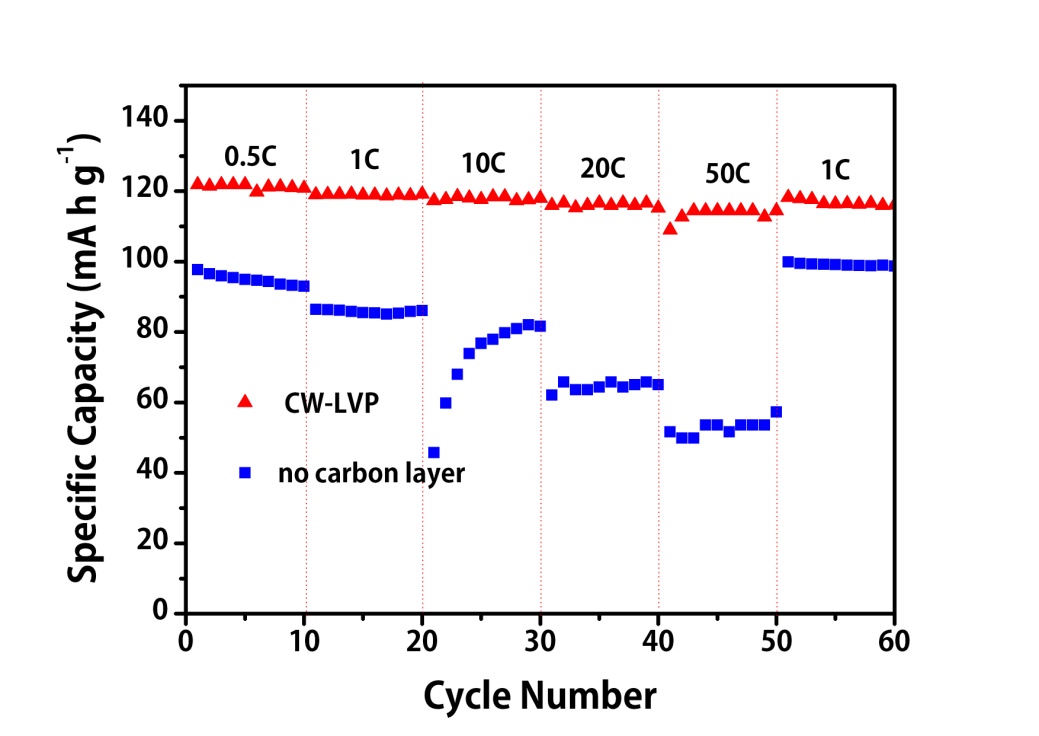


**Figure S10.** Rate performances of the CW-LVP microspheres and the LVP electrode without carbon layer.


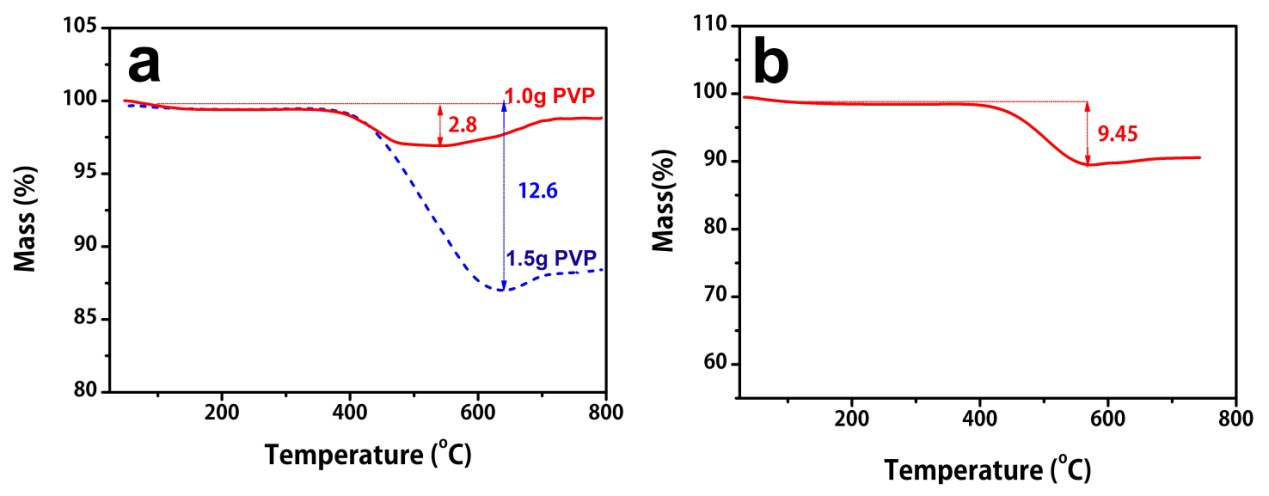


**Figure S11.** TG curves of the LVP samples (a) prepared using different amount of PVP: 1.0 g and 1.5 g; TG curve of the LVP microflowers (b) prepared by adding 10 mL distilled water in the solvothermal solution. The measurements were conducted in air using a temperature ramping rate of 10 ^o^C min^-1^.

**Table S1** The electrochemical properties comparison between CW-LVP and previously reported LVP cathodes.

| Structure | Current density | | Initial Capacity (cycle number) | | Final Capacity (cycle number) | | Retention rate | |  |
| --- | --- | --- | --- | --- | --- | --- | --- | --- | --- |
| **CW-LVP (this work)** | | 50 C | | 105.3 (1) | | ~85 (5000) | | 80.7% | |
| C/Li_3_V_2_(PO_4_)_3_ nanowires^39^ | | 5 C | | 120 (1) | | ~96 (3000) | | 80% | |
| C/Li_3_V_2_(PO_4_)_3_ composite^14^ | | 50 C | | ~90 (2) | | ~80 (2500) | | ~ 88.9% | |
| 3D foams^40^ | | 50 C | | 91 (1) | | — | | — | |
| C/Li_3_V_2_(PO_4_)_3_ nanoparticles ^46^ | | 20 C | | 83 (1) | | — | | — | |
| C/Li_3_V_2_(PO_4_)_3_ nanoparticles ^47^ | | 50 C | | 74.5 (1) | | ~67 (250) | | 90.8% | |
| C/Li_3_V_2_(PO_4_)_3_/graphene ^48^ | | 50 C | | 85 (1) | | — | | — | |
